# Maximum Entropy Modeling the Distribution Area of *Morchella* Dill. ex Pers. Species in China under Changing Climate

**DOI:** 10.3390/biology11071027

**Published:** 2022-07-08

**Authors:** Yu-Ting Cao, Zhao-Ping Lu, Xin-Yu Gao, Mi-Li Liu, Wei Sa, Jian Liang, Le Wang, Wei Yin, Qian-Han Shang, Zhong-Hu Li

**Affiliations:** 1 Key Laboratory of Resource Biology and Biotechnology in Western China, Ministry of Education, College of Life Sciences, Northwest University, Xi’an 710069, China; caoyuting0011@163.com (Y.-T.C.); 202032601@stumail.nwu.edu.cn (Z.-P.L.); 202032597@stumail.nwu.edu.cn (X.-Y.G.); liumili@stumail.nwu.edu.cn (M.-L.L.); 2 State Key Laboratory of Plateau Ecology and Agriculture, Qinghai University, Xining 810086, China; sawei3699@163.com (W.S.); liangjianws@126.com (J.L.); lelenmg@163.com (L.W.); yinweixzyn@126.com (W.Y.)

**Keywords:** *Morchella*, MaxEnt, geographic distribution, environmental factors, global warming

## Abstract

**Simple Summary:**

Climate change has always been a noticeable factor in the research of species distribution. In recent decades, the habitats of species have been gradually destroyed due to the changing climate. Thus, in order to predict how climate change will influence the survival and suitable habitats of wild *Morchella* Dill. ex Pers. species in China, we used a maximum entropy model to simulate the changes in its distribution area from historical periods to future periods. Our results illustrate that precipitation, elevation and temperature are indispensable factors affecting the presence and suitable habitats of wild *Morchella* species. Furthermore, this research showed us a promising trend that, regardless of which scenario, the suitable area of the species will increase to a certain scale in the near future. Based on these findings, we could explore and design an optimal scheme for the conservation of wild *Morchella* resources.

**Abstract:**

*Morchella* is a kind of precious edible, medicinal fungi with a series of important effects, including anti-tumor and anti-oxidation effects. Based on the data of 18 environmental variables and the distribution sites of wild *Morchella* species, this study used a maximum entropy (MaxEnt) model to predict the changes in the geographic distribution of *Morchella* species in different historical periods (the Last Glacial Maximum (LGM), Mid Holocene (MH), current, 2050s and 2070s). The results revealed that the area under the curve (AUC) values of the receiver operating characteristic curves of different periods were all relatively high (>0.83), indicating that the results of the maximum entropy model are good. Species distribution modeling showed that the major factors influencing the geographical distribution of *Morchella* species were the precipitation of the driest quarter (Bio17), elevation, the mean temperature of the coldest quarter (Bio11) and the annual mean temperature (Bio1). The simulation of geographic distribution suggested that the current suitable habitat of *Morchella* was mainly located in Yunnan, Sichuan, Gansu, Shaanxi, Xinjiang Uygur Autonomous Region (XUAR) and other provinces in China. Compared with current times, the suitable area in Northwest and Northeast China decreased in the LGM and MH periods. As for the future periods, the suitable habitats all increased under the different scenarios compared with those in contemporary times, showing a trend of expansion to Northeast and Northwest China. These results could provide a theoretical basis for the protection, rational exploitation and utilization of wild *Morchella* resources under scenarios of climate change.

## 1. Introduction

Climate change is an environmental factor that all organisms on Earth have to face all the time. With the changing climate, the spatial geographical distributions and distribution areas of species are also changing. In recent years, global climate change has resulted in shifts of the habitats of various species and even the extinction of some species [1,2]. Research has shown that some species will move to high-latitude and -altitude regions two to three times faster in the future [3]. Thus, scientifically evaluating and predicting the impact of climate change on species distribution and biodiversity have attracted great attention [4]. In order to understand the change characteristics of different species under future climate conditions, research on the relationship between species and climate is of great urgency.

A possible solution is to use the species distribution model (SDM). SDM is an important method for the analysis of changes in species distribution ranges, and it is widely used in biogeography studies. In recent years, using species distribution models to predict the real and potential distributions of endangered species [5], rare species [6] and invasive species [7] has become a hotpot in the field of ecology. In the research of species distribution, climate, soil and other factors (such as the migration ability of species) can influence species’ geographical distributions to some extent [8]. Combined with species distribution data and environmental factors, SDM projects these data to a certain geographical spatial range, and it also estimates the species’ suitable regions for survival and their living environment preferences [9,10,11]. The SDMs that are now available include BIOCLIM, the Ecological Niche Factor Analysis (ENFA), the Generalized Linear Model (GLM), the Bayesian Approach (BA), Genetic Algorithms (GAs) and MaxEnt [12]. Among these, MaxEnt uses the actual presence data of species and the corresponding environmental variables to calculate the ideal state of species distribution under certain niche constraints, that is, the possible distribution of species in the predicted area when the entropy is maximum. This model differs from other models in the requirement of the data of species distribution sites, the setting of model parameters and the handling of environmental variables [13,14]. Most models need the presence and absence data of species distribution; however, MaxEnt only relies on real existing sites [15]. The probability distribution of MaxEnt has a concise mathematical definition, which is easy for analysis. For instance, as with GLM and GAM, the additivity of the model makes it possible to interpret how each variable relates to suitability in the absence of interactions between variables [16]. At the same time, the prediction accuracy of the MaxEnt model is so high that it can reflect the probability of the occurrence of species correctly to a certain degree under the circumstance of small sample size [17].

In view of the superiority of MaxEnt, many scholars have published a series of significant research achievements using MaxEnt, providing a highly valuable theoretical basis to various fields, such as the management of invasive species, the protection of biodiversity and the selection of species living environmental conditions. Not only can MaxEnt be used for plants and animals, but it can also be used for fungi. Sun et al. used MaxEnt to simulate the suitable habitat of giant pandas and explained the responses of the species to environmental variables at different scales [18]. Liu et al. simulated the distribution of *Houttuynia cordata* Thunb (Ceercao) under current climate conditions and predicted its potential geographical distribution changes, and the results revealed that the area of suitable habitat of Ceercao decreased under three scenarios of greenhouse gas emissions in the 2050s and 2070s [19]. Yuan et al. predicted the potential distribution of *Phellinus baumii* Pilát, *Phellinus igniarius* (L.) Quél. and *Phellinus vaninii* Ljub. and found that the accuracy of the results was high [20].

*Morchella* is a group of important fungi belonging to the Morohellaceae of Ascomycotina, which is widely distributed in the northern hemisphere. Because of its rich nutrition and medicinal values, *Morchella* species occupy a place in the most precious edible fungi and attract the attention of many mycologists [21]. The natural bioactive components [22], such as polysaccharides, proteins and lipids, extracted from *Morchella* play a significant role in disease prevention, including immune regulation [23], anti-tumor activities [24] and anti-oxidation activities [25]. Molecular phylogenetic studies have shown that *Morchella* can be divided into three main evolutionary clades, namely, Yellow *Morchella*, Black *Morchella* and Red *Morchella* [26]. East Asia and China are the possible differentiation and diversity centers of *Morchella* species. At present, more than thirty species of *Morchella* have been recorded in China [27], which is one of the countries with the most abundant wild *Morchella* resources.

However, overexploitation and habitat destruction pose a severe threat to the species diversity of wild *Morchella* [28]; meanwhile, the specific requirements and environmental qualities of *Morchella* growth have been long discounted in the field of mycology [29]. Additionally, Taheri et al. [2] showed that there were few studies on the geographical range of fungi related to climate change in comparison with those of plants and animals. At present, it is still unclear how climate change will impact the geographical distribution of *Morchella* species in different time periods. This study aims to predict the potential distribution of *Morchella* species under different scenarios of historical and future climates based on a MaxEnt model. The purpose of this study is to analyze the effect of environmental factors on the formation of *Morchella* fruiting bodies and to simulate the changes in the potential distribution areas of *Morchella* in the different periods. It is expected that the results will provide a scientific foundation for the biodiversity and wild resource conservation of *Morchella* in the future.

## 2. Materials and Methods

### 2.1. Source of Species Distribution Data

The occurrence data of *Morchella* species were acquired from field surveys and published papers; we obtained a total of 288 sites. First, repeated locations were discarded, and then the buffer method was used. The spatial resolution of environmental factors was 2.5 arcminutes, and spatially coincident data points within 5 km of each other were discarded, allowing for model overfitting caused by duplicated distribution sites to be avoided. Finally, a total of 180 sites of *Morchella* were retained (Figure S1 and Table S1).

### 2.2. Environmental Factor Acquisition and Pretreatment

A total of 19 environmental factors (Bio1–Bio19, Table 1) were downloaded from the World Climate Database (http://www.woldclim.org/, accessed on 9 October 2021), and the spatial distribution rate was 2.5 arcminutes. A total of 2 terrain variables and 7 soil variables (Table 1) were obtained from the Harmonized World Soil Database (HWSD, http://www.fao.org/soils-portal/, accessed on 9 October 2021). Terrain and soil variables were jointly determined by the Food and Agriculture Organization of the United Nations, the International Institute for Applied Systems Analysis, the Institute of Soil Science, the Chinese Academy of Sciences and the European Commission’s joint research center. The spatial resolution of these data was unified into 2.5 arcminutes, and the data were all transformed into ASCII format using ArcGIS 10.2. The potential distributions of *Morchella* were assessed over five periods, namely, the Last Glacial Maximum (LGM), Mid Holocene (MH), current, 2050s and 2070s. Both the past and future climatic data adopt the CCSM4.0 model published in the IPCC Fifth Report; we selected three different scenarios of greenhouse gas emissions for the future periods (Table S2), and these scenarios are defined according to the resulting total radiative forcing in 2100 [30].

**Figure 1:**
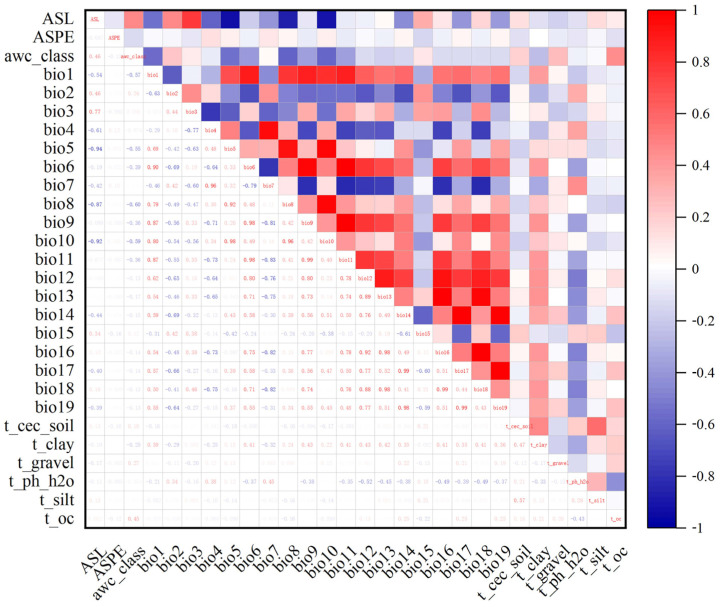
Correlation analysis of various environmental factors. The data in the lower left half of the graph represent the correlation coefficient values between environmental variables. Red represents positive correlations, and blue represents negative correlations. The deep red and blue represent higher correlation coefficients between two variables. The explanations of the variables are provided in Table 1.

To avoid the overfitting of the results due to the high collinearity of environmental variables [31], environmental variable contribution and correlation analyses were performed based on MaxEnt and SPSS programs. We used the MaxEnt 3.4.1 program (http://biodiversityinformatics.amnh.org/open_source/maxent/, accessed on 9 October 2021) to analyze the variables’ contributions based on environmental variables and the distribution sites of *Morchella*, and we set the repetitions to 10 times. Next, the information on the environmental factors of *Morchella* was extracted using ArcGIS 10.2, and a Pearson correlation analysis was performed between environmental variables in SPSS 25 (Figure 1). Combined with the contribution of environmental factors, we retained factors with correlation coefficients under 0.8 with regard to the top six factors. A couple of environmental factors had correlation coefficient values greater than |0.8|, and only one variable with a higher contribution was retained and used in the MaxEnt models [32,33]. Finally, 18 environmental factors were used in the modeling (Table 1).

### 2.3. MaxEnt Model Analysis

#### 2.3.1. Model Parameter Selection

The distribution sites of *Morchella* species and 18 environmental factors were imported into the MaxEnt3.4.1 program for a modeling analysis. A total of 25% of the distribution data were selected randomly as the testing set to examine model accuracy, and the remaining 75% were used as the training set [34]. We ran 10 bootstrap replicates, whose type was Subsample. Apart from this, the threshold selected was maximum training sensitivity plus specificity, the output format was Cloglog, and the other parameters were left as their defaults.

#### 2.3.2. Model and Environmental Variable Evaluation

AUC is a comprehensive criterion that represents the accuracy and specialty of ROC. It was first introduced into the evaluation of species distribution model accuracy in 1997 [35], and since then, it has been used to evaluate models’ performances. The value of AUC ranges from 0.5 to 1. If the value is closer to 1, it means that the predictive precision of the model is higher. An AUC value under 0.7 indicates that the simulation effect of the model is poor; an AUC value between 0.7 and 0.8 indicates that the simulation effect of the model is moderate; an AUC value between 0.8 and 0.9 indicates that the simulation effect of the model is good; and certainly, when the AUC value is higher than 0.9, the simulation effect is excellent [36]. In addition, MaxEnt provides a Jackknife method to analyze the relative contribution and importance of environmental variables on *Morchella* and to determine the major environmental factors.

#### 2.3.3. Suitable Region Classification

According to the assessment of presence probability in the IPCC Fifth Report [37], we reclassified the suitable habitat of *Morchella* using the Reclass module in ArcGIS 10.2 with the natural segment method. The habitat of *Morchella* was divided into four grades using the natural segment method: unsuitable habitat (0 ≤ value ≤ 0.13), low suitable habitat (0.13 < value ≤ 0.35), moderate suitable habitat (0.35 < value ≤ 0.63) and high suitable habitat (0.63 < value ≤1).

#### 2.3.4. Change in Distribution Center of *Morchella*

SDM tools is a GIS toolkit used in analyzing the centroid change in suitable distribution regions [38]. In this study, SDM tools and the binary suitable areas of *Morchella* in different periods were used to calculate the geographical location of its distribution center, thus illustrating the route of the temporal and spatial evolution of *Morchella*.

## 3. Results

### 3.1. Evaluation of the Accuracy of the Model

As shown in the AUC value of the ROC curve operated by MaxEnt, the average AUC value of the training data of the *Morchella* potential distribution model under past climatic conditions was 0.907, and the mean AUC value of the test data was 0.847; under the current period, the average AUC value of the training data was 0.905, and the mean value of the test data was 0.852; as for the future periods, the average AUC value of the training data was 0.903, and the mean value of the test data was 0.848 (Table 2). According to the evaluation standard of the AUC value, these results are good and reliable.

### 3.2. Dominant Environmental Factors

Table 3 shows the relative contribution of modeling environmental factors. Bio17, elevation, Bio11 and Bio1 were the main environmental factors affecting *Morchella* distribution. In the LGM, MH, current and future periods (2050s and 2070s), the cumulative contribution rates reached 75.8%, 79.9%, 70.6%, 74.6%, 77.5%, 74.6%, 76.8%, 74.0% and 80.0%. Bio17 affected *Morchella* the most, and elevation and Bio11 were the second and third most effective factors, respectively, which also had a great impact on the probability of *Morchella* occurrence.

**Figure 2:**
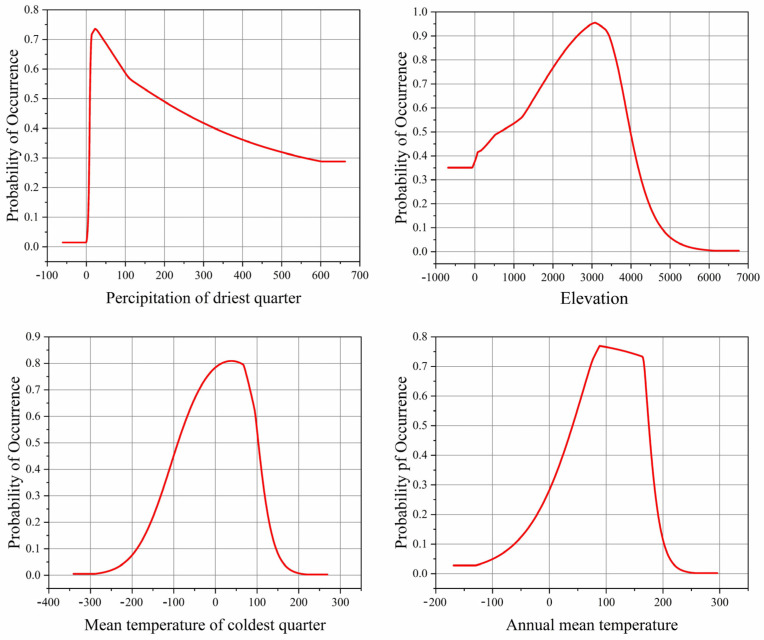
Single-factor response curves to the main environment factors.

Based on the single-factor response curves, the influence of dominant factors on *Morchella* presence probability was analyzed. The high suitable environmental conditions for the survival of *Morchella* were as follows: Bio17 was 11.32–77.78 mm, elevation was 1480.78–3827.03 m, Bio11 was −5.98–9.32 °C and Bio1 was 6.16–16.98 °C (Figure 2).

### 3.3. Potential Geographical Distribution and Evaluation of Suitable Areas of Morchella

#### 3.3.1. Suitable Areas in the Past

The suitable habitat in both LGM and MH decreased compared to that of the present age. MaxEnt predicted that the total suitable area of *Morchella* decreased by 12.43% in LGM and that the high suitable area decreased by 5.07%, which were mainly reflected in the reduction in the suitable area in the southeast of Gansu, the center and south of Shaanxi, and the north of Guizhou in China; the moderate suitable area decreased by 2.48%, which was predicted to mainly occur in XUAR and North China; and the low suitable area decreased by 4.88%, mainly in Northeast China and the northwest of XUAR (Figure S2, and Table 4 and Table 5).

As for MH, the reduction range was smaller than that in LGM. There was slight disparity in MH and current, which we could not clearly distinguish in the figures (Figures S2 and S3). More details about the distribution area are shown in Table 4 and Table 5.

#### 3.3.2. Suitable Areas of Current Times

It can be seen in Figure S3 that the suitable habitat of *Morchella* is relatively extensive under contemporary climate conditions. The total suitable habitat area was approximately 405.8195 × 10^4^ km^2^, accounting for 42.34% of China’s territorial area (Table 4 and Table 5). It was largely located in Southwest and Northwest China, covering northern Yunnan, southeast Tibet, Sichuan, central and southern Shaanxi, southern Shanxi, northern Guizhou, southeast Gansu, northwest Xinjiang and some parts of Fujian.

#### 3.3.3. Evaluation of Potential Distribution Areas of *Morchella* in the Future

In the 2050s, the total suitable area of *Morchella* showed an increasing trend. Under the three different scenarios, it increased by 3.88%, 4.93% and 4.69%, respectively (Table 5), and the area amplification amplitude first increased and then decreased with the increase in greenhouse gas emissions. Although the total suitable area in the 2070s also increased, the area amplification amplitude raised with the increase in greenhouse gas emissions. The potential distribution areas were simulated to be increased by 2.10% (RCP2.6), 6.04% (RCP4.5) and 6.71% (RCP8.5).

**Figure 3:**
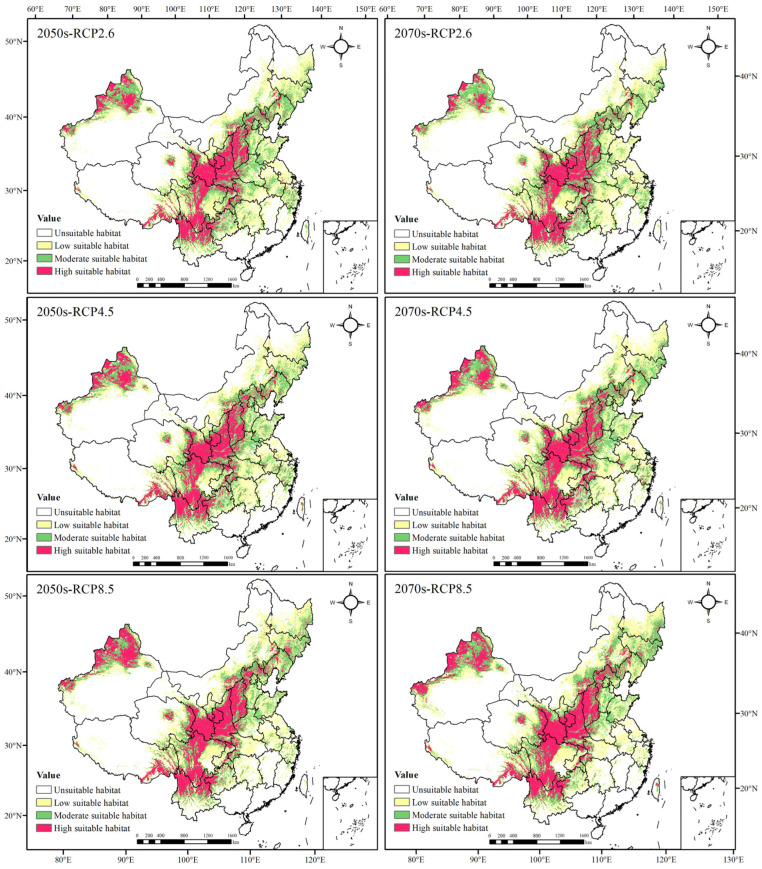
Prediction of potential geographical distributions of *Morchella* in different climatic scenarios.

The potential geographical distributions of *Morchella* in the 2050s and 2070s differed from contemporary times under the three scenarios. The low suitable area and the high suitable area increased, showing an expansion trend. Among them, the increase in the low suitable regions was mainly reflected in Heilongjiang, Jilin and Inner Mongolia autonomous regions, and the increase in the high suitable regions was primarily located in XUAR and Shanxi Province. The area of the moderately suitable habitat had little change, presenting a decreasing trend in the 2050s with the increase in greenhouse gas emissions, whereas it first expanded and then degraded in the 2070s (Figure 3, and Table 4 and Table 5).

### 3.4. Possible Influence of Climate Change on the Geographic Distribution of Morchella

**Figure 4:**
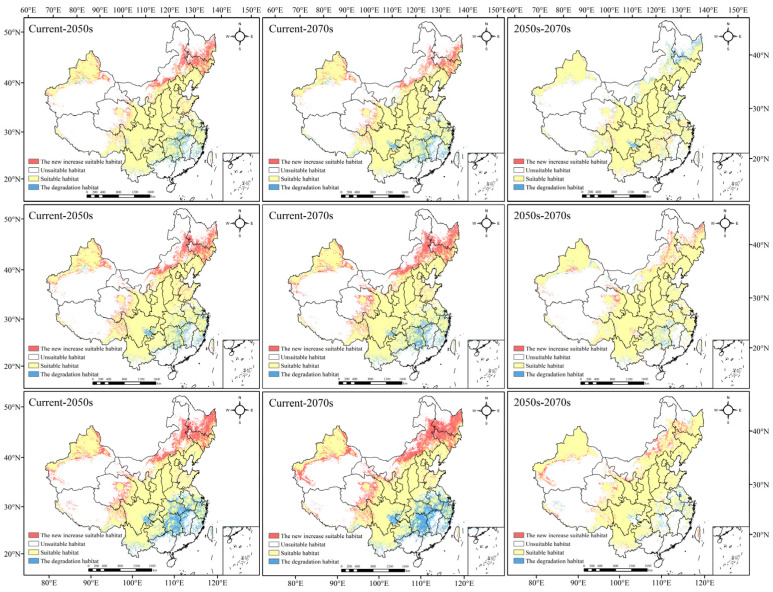
Changes in the suitable habitat of *Morchella* under climate change (the scenario of the first line is RCP2.6, the scenario of the second line is RCP4.5, and the scenario of the third line is RCP8.5).

Figure 4 and Figure S4 show the temporal and spatial evolution of the geographical distribution of *Morchella* under different periods and emission levels (Figure 4). In the future periods, the distribution area of *Morchella* showed an overall growing trend, but the growth range was slightly different.

In the RCP2.6 scenario, the total suitable area increased in the 2050s and 2070s compared with that in contemporary times, but the expansion area in the 2050s was greater than that in the 2070s. The suitable habitat would expand to Northeast China, Inner Mongolia, Qinghai and XUAR. Most of the suitable areas in Hunan, Jiangxi and Fujian would decrease, and the suitable areas in Sichuan, Guizhou, Hubei, Jiangsu, Zhejiang and Anhui would also degrade to a certain extent. From the 2050s to the 2070s, the suitable area of *Morchella* declined, and the degradation areas were mainly in Heilongjiang, Jilin and Sichuan Provinces.

In the RCP4.5 scenario, the expansion and degradation trends of the *Morchella* suitable area in the 2050s and 2070s were roughly the same as those in the RCP2.6 scenario. However, there was a difference in the newly added and degraded areas from the 2050s to the 2070s compared to those in the RCP2.6 scenario. In addition to the area of suitable habitat in Heilongjiang, Xinjiang, Qinghai and Inner Mongolia, which would increase significantly, Gansu, Sichuan and Tibet also showed an expansion trend. The degradation area was mainly concentrated in Hunan and Hubei; small parts of Anhui, Yunnan, Guizhou and XUAR would degrade.

In the RCP8.5 scenario, the newly added areas in the 2050s and 2070s were mainly located in Jilin, Heilongjiang and Inner Mongolia, and they were significantly larger than those in the first two scenarios; degradation was roughly the same as that in the first two scenarios, but the scope of degradation doubled. From the 2050s to the 2070s, the newly added suitable habitat was concentrated in Xinjiang and northwest Gansu, central Inner Mongolia and southwest Heilongjiang; the degradation in habitat was mainly in Hunan, central Anhui, Southeast Sichuan and some coastal areas in the southeast.

To sum up, the suitable areas of *Morchella* generally showed an expansion trend to most of Northeast China and a small part of Northwest China under different scenarios; additionally, large-scale degradation would happen in Central South and Southeast China, indicating that high-latitude areas may be more suitable for *Morchella* to survive in a warming climate.

### 3.5. Change in Morchella Distribution Center of Suitable Areas

**Figure 5:**
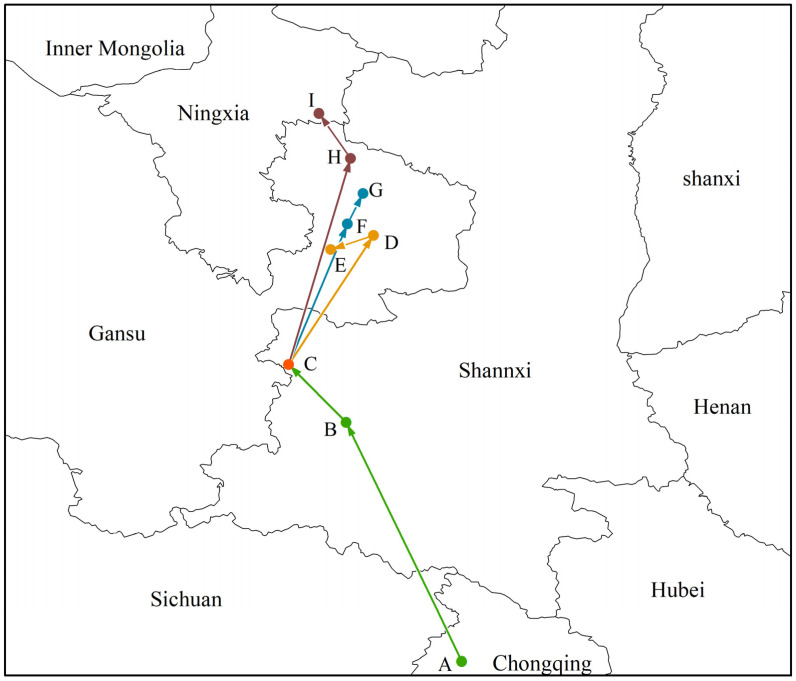
Migratory routes of potential distribution centers of *Morchella* in the context of climate change. Among them, the meaning of the letters were (A) LGM, (B) MH, (C) current, (D) 2050s—RCP2.6, (E) 2070s—RCP2.6, (F) 2050s—RCP4.5, (G) 2070s—RCP4.5, (H) 2050s—RCP8.5, (I) 2070s—RCP8.5.

In this study, the geographical coordinates of *Morchella* distributions in different periods were calculated using SDM tools. The results show that the current distribution center of *Morchella* is located in the southwest of Shaanxi Province (point C, 106°67′ E, 34°48′ N). From the LGM to the MH, its geographical distribution center moved from the north of Chongqing (point A, 108°56′ E, 31°25′ N) to the southwest of Shaanxi Province (point B, 107°30′ E, 33°85′ N), and the migration distance was 305.70 km. In the future, regardless of which scenario, the distribution centroid of *Morchella* would migrate to the northeast to Gansu Province or even to Ningxia Province (Figure 5).

## 4. Discussion

The geographic distribution and richness of species can be influenced by climate, soil and other environmental factors. *Morchella* is a group of low-temperature aerobic fungi, whose growth and spatial distribution may be restricted by changes in temperature, light and humidity [39]. Furthermore, environmental characteristics, such as soil type, pH and the availability of nutrients and water in the substrate, are also the main factors affecting the formation of *Morchella* fruit bodies [29]. As MaxEnt has certain advantages in fungi modeling [40,41], this study discussed the dominant environmental factors affecting *Morchella* and its potential suitable habitats using the model.

### 4.1. Change in Geographic Distributions

Since the 1950s, global warming has accelerated, greenhouse gas emissions have increased, and the sea level has risen [42]. In order to study the effect of climate change on the geographic distributions of *Morchella*, three different greenhouse gas emission scenarios (RCP2.6, RCP4.5 and RCP8.5) of the CCSM 4.0 model in the IPPC Fifth Report were selected as the climate variables. Since there was little difference between RCP4.5 and RCP6.0, we chose RCP4.5 [43]. The results show that different emission scenarios had a certain impact on the geographical distribution of *Morchella*. Cao et al. [38] simulated the migration of the suitable distribution areas of *Zelkova serrata* in China under different climatic scenarios; the results showed that the suitable areas of this species decreased significantly in Guangdong, Yunnan, Guangxi and Hainan and that its distribution would move to the northeast as the climate becomes warmer. Based on 89 effective distribution sites of *Artemisia ordosica* and 19 bioclimatic factors, Lu et al. [44] predicted that the center of the potential distribution areas of *Artemisia ordosica* lied in Mu Us Desert under future climate conditions, with a trend of expansion to Northeast China (Jilin, Heilongjiang, Liaoning and some parts of Hebei). Pan et al. [45] predicted the suitable distribution areas of two *Litsea coreana* species, namely, *Litsea coreana* Levl. Var. *sinensis* and *Litsea coreana* Levl. Var. *lanuginosa*, in China, and they indicated that the overall suitable habitat area would increase slightly in the future and migrate to high-latitude and -altitude areas compared with the current climate conditions. Similar to other species, the potential geographical distribution of *Morchella* in the future would also move to Northeast China. These results are consistent with the notion that some species will migrate to higher altitude and latitude regions in order to adapt to the environment with future climate warming [46].

As far as fungi are concerned, the distribution and change trends of each species’ suitable areas are different. The research conducted by Yuan et al. [20] showed that the most suitable survival areas of *Phellinus. baumii, Phellinus. igniarius* and *Phellinus. vaninii* were located in the northeast (Liaoning, Jilin and Heilongjiang), east, southwest (Sichuan, southeast Tibet and northwest Yunnan) and northwest (southwest Shaanxi and South Gansu) of China, highly overlapping with the distribution of *Morchella*. Wei et al. [40] analyzed the current and future geographical distribution patterns of *Ophiocordyceps sinensis* based on MaxEnt using climate, soil, altitude and other data, and they suggested that its habitat was mainly located in the Qilian Mountains, south Ganzhou of Gansu, the Aba Prefecture of Sichuan, northwest Yunnan, Qinghai (Yushu, Guoluo Prefecture) and east–central Tibet; apart from this, the geographical distribution of *Ophiocordyceps sinensis* showed a degradation trend under different greenhouse gas emission scenarios in the future, which is very different from the results of this study.

### 4.2. Climate Effects

The contribution and importance of environmental variables in the distribution of *Morchella* species slightly differed in the different historical periods. Contribution considers the correlations between environmental variables but importance does not [47]. The results of this study show that precipitation, altitude and temperature are the main environmental factors affecting the geographical distribution of *Morchella*, and these results are consistent with the research results indicating that humidity and temperature are important environmental factors affecting the geographical distribution and existence probability of *Batrachochytrium dendrobatidis* [48]. With the Jackknife method, the impact of environmental factors on *Morchella* was analyzed. The total contribution of variables related to precipitation accounted for 36.5%, the total contribution of variables related to temperature accounted for 31%, and the contribution of altitude was 22.5%. Among them, the most suitable value of Bio17 for the survival of *Morchella* was not less than 22.15 mm, the altitude was about 3082.19 m, Bio11 was about 3.84 °C, and Bio1 was about 8.86 °C (Figure 2). If the value of these factors are too high or too low, they will affect the survival probability of *Morchella*. The results further indicate that *Morchella* is a kind of hygrophilous, low-temperature fungi, preferring a higher altitude environment, which is consistent with relevant studies [49]. At the same time, when the temperature is low, there are fewer miscellaneous bacteria and pathogens, which is conducive to the growth and development of *Morchella*. However, this study regarded altitude as an independent variable, and it did not consider the relationship between altitude and climatic variables, such as temperature and precipitation. Research has shown [50] that temperature, precipitation and other climatic variables are suitable at global scales and meso-scales. Terrain variables such as altitude likely affect species distribution at meso-scales. Thus, the correlations between altitude and some climatic factors, which vary over space and time, should be further explored.

The protection of wild mushroom species has always been a serious problem. *Morchella* species have abundant benefits, and commercial markets and recreational pickers regard their fruit bodies as rare economic resources. Currently, fungi experts are exploring the suitable conditions for the growth of *Morchella* species. Mihail et al. [51] reported that the seasonal lengths of *Morchella* fruiting bodies were positively correlated with soil warming, showing that the optimal soil temperature in a narrow range was conducive to the explosive production of fruiting bodies. Further research has shown that vegetation type and the interaction between *Morchella* and vascular plants are intimately related with the distribution of *Morchella* species [29,51,52]. To increase the scale of *Morchella* species as much as possible and to develop wild *Morchella* resources sustainably, the protection of high suitable areas of *Morchella* should never be overlooked. Since the impact of human activities on species is difficult to measure and the relationship between species is hard to quantify, this study did not consider the effects of human activities and interspecific interactions on the geographical distribution of *Morchella*.

### 4.3. Limitation

The distributions of *Morchella* species show markedly high levels of continental endemism and provincialism in the northern hemisphere. Some research has shown that their distribution may be limited by dispersal [53]. On the one hand, they cannot expand via long-distance dispersal (LDD) due to the fact that, if the haploid colonies germinated by their ascospores have no chance to meet with the colonies of the opposite mating type, they would be unable to form fruit bodies. Additionally, *Morchella* species produce thin-walled mitotic spores, which are poorly adapted to LDD. On the other hand, the distribution of *Morchella* species may be highly related to human-mediated dispersal. This study did not consider these factors, such as dispersal restriction and human activities, and the range of environmental conditions simulated in this study may be different to actual conditions.

## 5. Conclusions

Based on the MaxEnt program, this study predicted the distribution and shift of the potential suitable habitats of *Morchella* in different periods. The results show that the model could simulate the distribution range of *Morchella* in China well. Environmental factors, such as Bio17, elevation, Bio11 and Bio1, had a relatively great impact on the survival and distribution of the genus *Morchella*. Currently, the total suitable area of *Morchella* species in China is 405.8195 × 10^4^ km^2^. In the 2050s and 2070s, the suitable areas would expand and migrate to Northeast and Northwest China. In addition, MaxEnt can simulate the suitable habitat of species under climate changing conditions, but it does not consider whether species can catch up with the speed of climate change [54,55]. Therefore, adding the migration process of species to the model in future research would overcome the above problems and more accurately simulate the dynamic process of species changes with the environment or climate [56].

## Figures and Tables

**Table 1:** Environmental factors used in this study. The highlighted cells are the environmental factors used in the modeling.

Abbreviation	Description	Unit
ASL	Elevation	m
ASPE	Aspect	°
awc_class	AWC range	Code
Bio1	Annual mean temperature	°C
Bio2	Mean diurnal range	°C
Bio3	Isothermality (Bio2/Bio7) (×100)	%
Bio4	Temperature seasonality (standard deviation ×100)	°C
Bio5	Max temperature of warmest month	°C
Bio6	Min temperature of coldest month	°C
Bio7	Temperature annual range (Bio5-Bio6)	°C
Bio8	Mean temperature of wettest quarter	°C
Bio9	Mean temperature of driest quarter	°C
Bio10	Mean temperature of warmest quarter	°C
Bio11	Mean temperature of coldest quarter	°C
Bio12	Annual precipitation	mm
Bio13	Precipitation of wettest month	mm
Bio14	Precipitation of driest month	mm
Bio15	Precipitation seasonality	mm
Bio16	Precipitation of wettest quarter	mm
Bio17	Precipitation of driest quarter	mm
Bio18	Precipitation of warmest quarter	mm
Bio19	Precipitation of coldest quarter	mm
t_ph_h2o	Topsoil pH (H_2_O)	−log (H+)
t_oc	Topsoil organic carbon	% weight
t_cec_soil	Topsoil CEC (soil)	cmol/kg
t_clay	Topsoil clay fraction	%wt.
t_gravel	Topsoil gravel content	%vol.
t_silt	Topsoil silt fraction	%wt.

**Table 2:** Prediction validation with ROC in MaxEnt.

	LGM	Mid Holocene	Current	2050s			2070s		
				RCP2.6	RCP4.5	RCP8.5	RCP2.6	RCP4.5	RCP8.5
Training data AUC	0.906	0.908	0.905	0.905	0.902	0.904	0.905	0.899	0.905
Test data AUC	0.849	0.844	0.852	0.842	0.856	0.841	0.839	0.849	0.858

**Table 3:** Percentage of environmental variables using the Jackknife method in different periods.

	LGM	Mid Holocene	Current	2050s			2070s		
				RCP 2.6	RCP 4.5	RCP 8.5	RCP 2.6	RCP 4.5	RCP 8.5
Bio17	33.9	33.2	31.2	33.4	33.6	33.6	32.3	31.5	32.3
ASL	20.7	18.4	19.7	18.9	17.1	19.2	20.7	20.0	19.7
Bio11	13.7	18.1	11.6	15.3	17.0	13.5	16.2	14.2	17.5
Bio1	7.3	10.2	8.1	7.0	9.8	8.3	7.6	8.3	10.5
Bio6	5.1	1.8	6.2	7.0	4.0	5.1	3.2	5.3	2.2
Bio12	2.5	1.7	5.4	1.9	2.1	2.0	3.4	3.7	3.2
Bio15	2.9	2.6	3.3	2.0	2.1	2.9	3.3	2.5	3.4
ASPE	3.1	3.0	3.2	3.4	3.5	3.0	2.8	3.4	3.0
t_gravel	2.4	2.2	2.2	1.9	2.1	2.0	2.2	2.1	1.8
t_ph_h2o	2.3	2.5	1.9	3.2	2.2	2.9	2.7	2.2	2.6
awc_class	1.6	0.9	1.7	1.5	1.3	1.1	1.1	1.8	1.2
t_cec_soil	0.9	1.0	1.3	1.1	1.2	1.2	0.7	0.7	1.4
Bio4	1.0	1.8	1.3	1.0	1.2	1.6	1.4	1.7	1.1
t_clay	0.7	0.9	0.9	0.7	0.9	0.9	0.7	0.8	0.9
t_silt	0.8	0.9	0.8	0.9	1.0	1.3	0.6	0.9	1.2
Bio3	0.8	0.5	0.7	0.7	0.5	1.1	0.6	0.6	0.7
t_oc	0.1	0.2	0.3	0.2	0.2	0.1	0.1	0.1	0.1
Bio2	0.1	0.2	0.1	0.2	0.2	0.2	0.1	0.3	0.2

**Table 4:** The area of each suitable region of *Morchella* in different periods.

Area of Each Suitable Habitat (The Change in Area Compared with Current, 10^4^ km^2^)					
Period		Low Suitable Habitat	Moderate Suitable Habitat	High Suitable Habitat	Total Suitable Habitat
LGM		136.3351 (−46.7500)	111.2656 (−23.8021)	39.1337 (−48.5330)	286.7344 (−119.0851)
Mid Holocene		162.0469 (−21.0382)	134.5938 (−0.4793)	92.0747 (+4.4080)	388.7154 (−17.1041)
Current		183.0851 (0.00)	135.0677 (0.00)	87.6667 (0.00)	405.8195 (0.00)
2050s	RCP2.6	195.4167 (+12.3316)	135.4514 (+0.3837)	112.1997 (+24.5330)	443.0678 (+37.2483)
	RCP4.5	198.1111 (+15.0260)	135.0417 (−0.0260)	120.0156 (+32.3489)	453.1684 (+47.3489)
	RCP8.5	191.5990 (+8.5139)	123.6476 (11.4201)	135.5119 (+47.8452)	450.7585 (+44.9390)
2070s	RCP2.6	197.7118 (+14.6267)	128.5017 (−6.5660)	99.7847 (+12.1180)	425.9982 (+20.1787)
	RCP4.5	200.8351 (+17.7500)	137.6337 (+2.5660)	125.2517 (+37.5850)	463.7205 (+57.9010)
	RCP8.5	196.1979 (+13.1128)	133.2396 (−1.8281)	140.7188 (+53.0521)	470.1563 (+64.3368)

Note: the figures in parentheses represent the change in area compared with current, and the units of all figures are 10^4^ km^2^.

**Table 5:** The percentage change in each suitable area of *Morchella* in different periods.

Area of Each Suitable Habitat (The Change in Area Compared with Current, %)					
Period		Low Suitable Habitat	Moderate Suitable Habitat	High Suitable Habitat	Total Suitable Habitat
LGM		14.22 (−4.88)	11.61 (−2.48)	4.08 (−5.07)	29.91 (−12.43)
Mid Holocene		16.90 (−2.20)	14.04 (−0.05)	9.61 (+0.46)	40.55 (−1.79)
Current		19.10 (0.00)	14.09 (0.00)	9.15 (0.00)	42.34 (0.00)
2050s	RCP2.6	20.39 (+1.29)	14.13 (+0.04)	11.70 (+2.55)	46.22 (+3.88)
	RCP4.5	20.67 (+1.57)	14.08 (−0.01)	12.52 (+3.37)	47.27 (+4.93)
	RCP8.5	19.99 (+0.89)	12.90 (−1.19)	14.14 (+4.99)	47.03 (+4.69)
2070s	RCP2.6	20.62 (+1.52)	13.41 (−0.68)	10.41 (+1.26)	44.44 (+2.10)
	RCP4.5	20.95 (+1.85)	14.36 (+0.27)	13.07 (+3.92)	48.38 (+6.04)
	RCP8.5	20.47 (+1.37)	13.90 (−0.19)	14.68 (+5.53)	49.05 (+6.71)

Note: the figures in parentheses represent the change in area compared with current, and the units of all figures are %s.

## Data Availability

The original data used in this study are available in Supplementary Materials.

## Supplementary Materials

The following are available online at https://www.mdpi.com/article/10.3390/biology11071027/s1. Figure S1: Recorded geographical distribution sites of Morchella in China, Figure S2: The geographical distribution of Morchella in LGM and MH periods, Figure S3: The current geological distribution of Morchella, Figure S4: The change in potential suitable habitat area of Morchella in different climatic scenarios, Table S1: Geographical distributions of Morchella species sampled in this study, Table S2: Types of RCPs and projected temperature increases.
