# Supplementary material for: Maximum Entropy Modeling the Distribution Area of Morchella Dill. ex Pers. Species in China under Changing Climate
Source: Biology (Basel). 2022 Jul 8;11(7):1027. doi: 10.3390/biology11071027 (PMC9312065; doi:10.3390/biology11071027)
Supplement: Supplementary file 1 [file biology-11-01027-s001.zip › biology-1747754-supplementary.pdf]

Table S1. Geographical distributions of *Morchella* species sampled in this study.

Table S2. Types of RCPs and projected temperature increases.

Figure S1. Recorded geographical distribution sites of *Morchella* in China.

Figure S2. The geographical distribution *Morchella* in LGM and MH period.

Figure S3. The current geographical distribution of *Morchella*.

Figure S4. The change in potential suitable habitat area of *Morchella* in different climatic scenarios.

Table S1. Geographical distributions of *Morchella* species sampled in this study.

Sample	Location	Longitude(°E)	Latitude(°N)	Elevation(m)
1	Leqing Zhejiang	121.12126	28.375913	
2	Longquan Zhejiang	119.21612	27.939284	
3	Gutian Zhejiang	118.752578	26.583262	
4	Putuo Zhejiang	122.396427	30.011219	
5	Dadong Yunnan	100.394814	27.146212	
6	Tongdian Lanping Yunnan	99.525078	26.692762	
7	Beiya Heqing Yunnan	100.18824	26.15349	
8	Judian Yunnan	99.651031	27.297022	
9	Jianchuan Yunnan	99.851837	26.316273	
10	Hutubi Xinjiang	86.883333	44.1913888	
11	Chabuchaer Xinjiang	81.32416	43.91689	
12	Fukang Xinjiang	87.9872222	44.1569444	
13	Shawan Xinjiang	85.1408333	43.9577777	
14	Shawan Xinjiang	85.8505555	44.2897222	
15	Shihezi Xinjiang	86.080555	44.2897222	
16	Shawan Xinjiang	85.628888	45.8763888	
17	Chabuchaer Xinjiang	81.313709	43.9166666	
18	Yanyuan Sichuan	100.806444	27.817961	2863
19	Yanyuan Sichuan	100.806194	27.818286	2885
20	Yanyuan Sichuan	100.806275	27.818422	2893
21	Yanyuan Sichuan	100.80645	27.818611	2904
22	Huidong Sichuan	102.545502	26.613497	1734
23	Mianning Sichuan	102.20643	28.356441	1837
24	Qianjiang Chongqing	108.771086	29.53322	
25	Xiaojin Sichuan	102.369426	31.002209	2911.7
26	Xuanhan Sichuan	107.7217	31.35386	1525.7
27	Beichuan Sichuan	104.30755	31.89148	1225.6
28	Qingchuan Sichuan	105.245407	32.581617	831.7
29	Jiuzhaigou Sichuan	104.23567	33.26142	2155.6
30	Kangping Liaoning	123.460277	42.963888	
31	Kangping Liaoning	123.225277	42.629166	
32	Kangping Liaoning	123.207777	42.581944	
33	Shangri-La Yunnan	99.46151	27.698362	
34	Taishan Shandong	117.391838	36.017612	
35	Taishan Shandong	117.097923	36.308891	
36	Zhuolu Hebei	115.211383	40.385834	
37	Kanasi Xinjiang	87.042951	48.700435	
38	Zhaosu Xinjiang	80.35	42.41	2630
39	Urumchi Xinjiang	86.59	43.3	1490
40	Shangri-La Yunnan	99.46	28.01	3128

41	Ranwu Xizang	96.4	29.29	3900
42	Lulang Xizang	94.44	29.46	3472
43	Banma Qinghai	100.49	32.88	3000
44	Minhe Qinghai	102.48	36.19	2746
45	Hongyuan Sichuan	102.36	32	1970
46	Tianzhu, Gansu	102.84	37.24	3354
47	Diebu Gansu	103.13	34.03	2387
48	Zhouqu Gansu	104.22	33.45	1369
49	Malu Gansu	103.31	34.5	2811
50	Zhamashi Qinghai	100.03	38.12	2744
51	Zhugu Qinghai	102.06	37.1	3100
52	Fangshan Shanxi	111.21	37.49	1619
53	Fu Shaanxi	109.22	35.59	938
54	Jiuzhaigou Sichuan	103.55	33.15	2435
55	Maerkang Sichuan	102.37	31.52	3358
56	Laojunshan Yunnan	99.27	27.11	2555
57	Altay Xinjiang	88.14023	47.84564	
58	Urumchi Xinjiang	88.31104	43.36378	
59	Shihezi Xinjiang	86.07893	44.30653	
60	Akxoki Xinjiang	82.98046	46.74532	
61	Wudu Gansu	104.92667	33.39189	
62	Kang Gansu	105.60855	33.32937	
63	Wen Gansu	104.68343	32.94396	
64	Cheng Gansu	105.74222	33.75062	
65	Zhouzhi Shaanxi	108.3415	33.9289333	993.5
66	Dayu Shaanxi	109.12695	33.9177833	1557.5
67	Mei Shaanxi	107.804166	34.0177	2010
68	Ningshan Shaanxi	108.491766	33.4793	2450.5
69	Ningshan Shaanxi	108.555783	33.6422	1181.5
70	Ningshan Shaanxi	108.793883	33.60805	1088.5
71	Shanyang Shaanxi	106.52666	31.632216	1100
72	Shangzhou Shaanxi	109.94083	33.84695	1005
73	Zhashui Shaanxi	109.542216	33.769716	1572
74	Mian Shaanxi	106.84555	33.338616	1950
75	Nanming Guizhou	106.759238	26.552863	
76	Huaxi Guizhou	106.676441	26.415879	
77	Xiaohe Guizhou	106.636577	26.653325	
78	Liupanshui Guizhou	104.837555	26.598833	
79	Wuchuan Guizhou	107.905456	28.569077	
80	Dejiang Guizhou	108.126411	28.270423	
81	Bijiang Guizhou	109.187435	27.696773	
82	Jiangkou Guizhou	108.8454	27.705922	
83	Shiqian Guizhou	108.230464	27.519722	
84	Yuping Guizhou	108.912578	27.241523	

85	Sinan Guizhou	108.259417	27.944548	
86	Baoshan Yunnan	98.703055	25.251944	3170
87	Baoshan Yunnan	98.461111	27.843055	2850
88	Baoshan Yunnan	98.833611	25.432777	1410
89	Baoshan Yunnan	98.690833	25.945833	1060
90	Linan Zhejiang	119.436458	30.353794	
91	Qingyuan Zhejiang	119.217434	27.757088	
92	Loufan Shanxi	111.79745	38.0672	
93	Jiaocheng Shanxi	111.483284	37.84269	
94	Fenyang Shanxi	111.78777	37.26542	
95	Xiangshan Zhejiang	121.721388	29.46	
96	Heshun Shanxi	113.57032	37.32963	
97	Xiaojin Sichuan	102.3644	30.99918	
98	Jiancaoping Shanxi	112.48699	37.94036	
99	Jiaocheng Shanxi	112.15478	37.55165	
100	Shouyang Shanxi	113.17666	37.89498	
101	Yushe Shanxi	112.97522	37.07084	
102	Wutai Shanxi	113.2554	38.72812	
103	Xiyang Shanxi	113.70718	37.61282	
104	Lingchuan Shanxi	113.28061	35.77512	
105	Shangri-La Yunnan	99.74317	27.84254	
106	Weixi Yunnan	99.341202	27.566418	
107	Fuwen Xinjiang	88.98046	46.74532	
108	Shangri-La Yunnan	99.79126	27.582403	
109	Hui Gansu	106.08785	33.76884	
110	Jianchuan Yunnan	99.8166666	26.6333333	
111	Barkam Sichuan	102.616666	31.866666	
112	Lueyang Shaanxi	105.883333	33.25	
113	Changji Xinjiang	87.2166666	44.366666	
114	Yulong Yunnan	99.45	27.2166666	
115	Fu Shaanxi	109.016666	35.866666	
116	Pengshui Chongqing	108.172578	29.299462	
117	Longyang Yunnan	99.243161	24.9689	
118	Longyang Yunnan	99.260234	25.160723	
119	Longyang Yunnan	98.847174	24.968641	
120	Zhouqu Gansu	104.37749	33.790815	
121	Luqu Gansu	102.494424	34.597087	
122	Xiahe Gansu	102.528577	35.208324	
123	Lintan Gansu	103.360533	34.698639	
124	Zhuoni Gansu	103.513616	34.594916	
125	Jiuzhaigou Sichuan	104.249547	33.257592	
126	Li Sichuan	103.173511	31.44255	
127	Jinchuan Sichuan	102.07061	31.482006	
128	Barkam Sichuan	102.213502	31.911748	

129	Kangding Sichuan	101.963555	30.004407
130	Ruoergai Sichuan	102.974391	33.584377
131	Luhuo Sichuan	100.682397	31.397992
132	Daowu Sichuan	101.13154	30.985326
133	Rangtang Sichuan	100.985583	32.271093
134	Xichang Sichuan	102.271484	27.900581
135	Wenchuan Sichuan	103.34628	30.880388
136	Wenchuan Sichuan	103.337201	30.904286
137	Wenchuan Sichuan	103.482754	31.123078
138	Wenchuan Sichuan	103.550688	31.539906
139	Wenchuan Sichuan	103.559224	31.591701
140	Yuexi Anhui	116.366555	30.855228
141	Taihu Anhui	116.315476	30.460113
142	Susong Anhui	116.135524	30.159787
143	Jinzhai Anhui	115.940441	31.733462
144	Xiao Anhui	116.953585	34.194269
145	Shucheng Anhui	116.955602	31.467692
146	Huocheng Xinjiang	80.885281	44.06225
147	Xinyuan Xinjiang	83.267022	43.435927
148	Gongliu Xinjiang	82.238226	43.488486
149	Zhaosu Xinjiang	81.137378	43.163497
150	Nilka Xinjinag	82.518008	43.804595
151	Pingquan Hebei	118.654232	41.239373
152	Shuangqiao Hebei	117.8862	41.036891
153	Pingquan Hebei	118.780345	41.226422
154	Pingquan Hebei	118.52472	41.207198
155	Pingquan Hebei	118.707427	41.023756
156	Yuanping Shanxi	112.717487	38.736476
157	Jingle Shanxi	111.945432	38.365147
158	Xinzhou Shanxi	112.740624	38.422383
159	Fangshan Shanxi	111.25037	37.90102
160	Zuoquan Shanxi	113.385452	37.088555
161	Deqin Yunnan	99.309807	27.803802
162	Deqin Yunnan	98.873475	28.300246
163	Deqin Yunnan	98.691677	28.726924
164	Sunan Gansu	98.550839	39.209378
165	Hunnan Liaoning	123.64983	41.942101
166	Heishan Liaoning	122.132926	41.659497
167	Chaoyang Liaoning	120.39619	41.503745
168	Jianping Liaoning	119.64938	41.409456
169	Xinmin Liaoning	122.842529	41.991513
170	Qingyuan Liaoning	124.93036	42.106761
171	Wafangdian Liaoning	121.554961	39.521578
172	Pingjiang Hunan	113.587604	28.707564

173	Liuyang Hunan	113.649398	28.169832
174	Fenghuang Hunan	109.605527	27.953848
175	Yuanling Hunan	110.402659	28.460358
176	Fuyang Zhejiang	119.797368	30.009596
177	Gucheng Yunnan	100.22549	26.87721
178	Gucheng Yunnan	100.264084	26.700333
179	Hami Xinjiang	93.51465	42.82699
180	Xiaoshan Zhejiang	120.235462	30.187587

Table S2. Types of RCPs and projected temperature increases.

Scenario	Radiative forcing	Projected temperature increases
RCP2.6	Decrease after the peak (3W/m^2) before 2100	1.6-3.6℃ ℃/2.4 ℃
RCP4.5	Stable at 4.5W/m^2 after 2100	2.4-5.5 ℃/3.6 ℃
RCP8.5	8.5W/m^2 in 2100	4.6-10.3 ℃/6.9 ℃

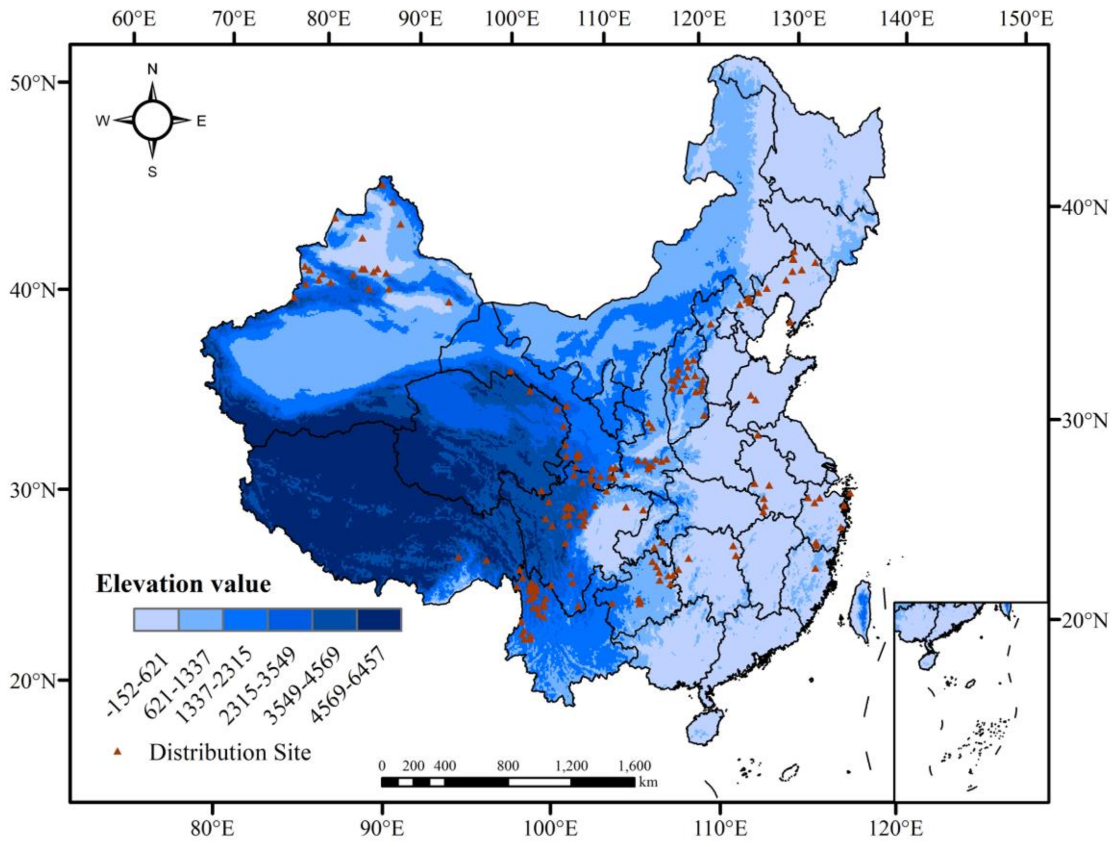

Figure S1. Recorded geographical distribution distribution sites of *Morchella* in China.

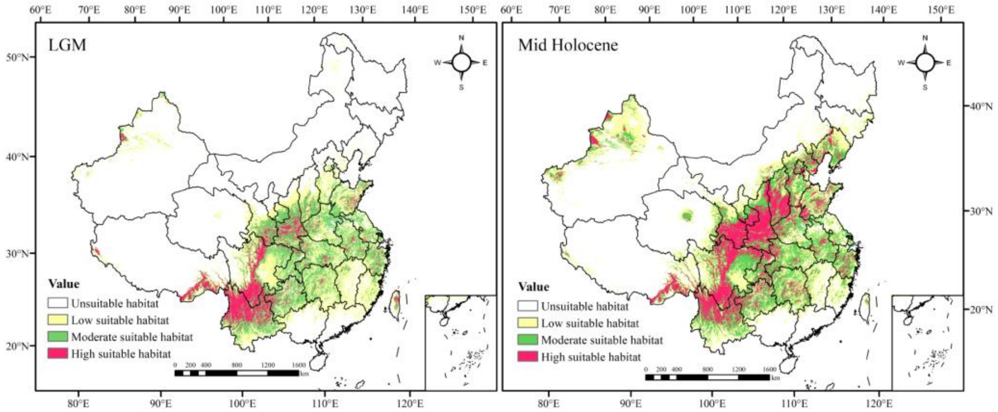

Figure S2. The geographical distribution *Morchella* in LGM and MH period.

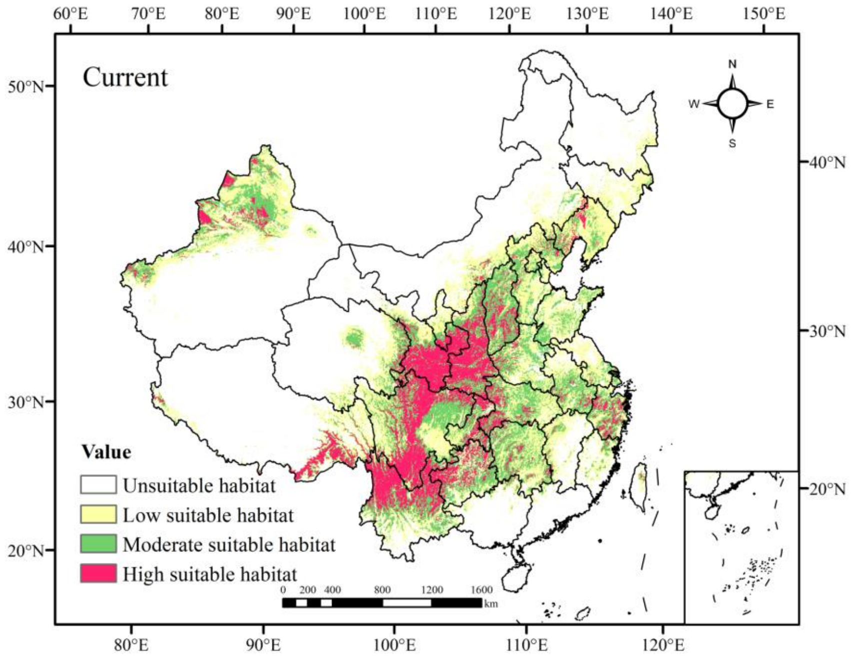

Figure S3. The current geographical distribution of *Morchella*.

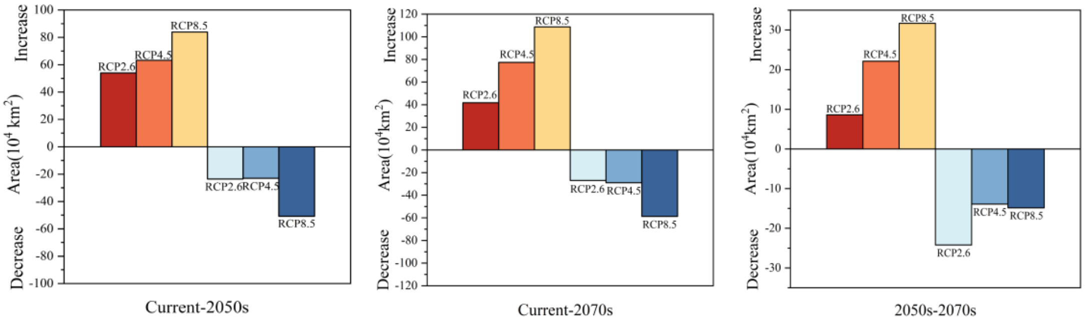

Figure S4. The change in potential suitable habitat area of *Morchella* in different climatic scenarios.
